# Exploring Heterogeneity of Longevity: Insights From the Health Retirement Study

**DOI:** 10.1093/geroni/igaf122.069

**Published:** 2025-12-31

**Authors:** Yeon Ji Ryou, Peter Martin

**Affiliations:** Iowa State University, Ames, Iowa, United States; Iowa State University, Ames, Iowa, United States

## Abstract

Understanding the heterogeneity in longevity among oldest-old adults is crucial for identifying distinct pathways to extended life, as aging experiences vary by individuals. This study aimed to identify unique longevity profiles among adults 80 years and older using the 2020 RAND Health and Retirement Study (HRS) data. A complete sample of 5,436 participants was selected (M = 86.09). Latent profile analysis (LPA) was conducted using indicators of respondents’ and parental longevity, physical health (chronic conditions, activities of daily living limitations), cognitive function, personality traits (neuroticism and conscientiousness), and psychological well-being (life satisfaction). Four distinct longevity profiles were identified: (1) Burdened Survivors (n = 483; 8.89%), experiencing chronic diseases, ADL limitations, low conscientiousness, relatively high neuroticism, and lower life satisfaction but with more prolonged survival; (2) Goldilocks survivors (n = 1,143; 21.03%), with moderate longevity, average well-being, and neither the best nor worst outcomes; (3) Thriving Short-Lived Survivors (n = 3,276; 60.27%), enjoying excellent overall well-being but with the shortest lifespan; and (4) Longevity at a Cost (n = 534; 9.82%), living the longest but enduring significant physical and psychological challenges. The Cox proportional hazard model subsequently assessed mortality risk across profiles. There are significant differences in mortality risk across profiles, with Thriving Short-lived Survivors exhibiting the highest mortality risk, showing a 123.4% increased risk of death compared to the reference group (Longevity at a Cost). These findings highlight the importance of understanding varied longevity trajectories to develop tailored and nuanced interventions that address physical and psychological challenges while fostering resilience and enhancing the quality of life among the oldest-old population.

